# Preferential phosphodiesterase inhibition as a novel strategy to modulate key pathways in systemic sclerosis

**DOI:** 10.3389/fimmu.2026.1829186

**Published:** 2026-10-02

**Authors:** Jörg H. W. Distler, Tracy Frech, Zsuzsanna H. McMahan

**Affiliations:** 1 Department of Rheumatology, University Hospital Düsseldorf, Heinrich Heine University, Düsseldorf, Germany; 2 Hiller Research Center, University Hospital Düsseldorf, Heinrich Heine University, Düsseldorf, Germany; 3 Division of Rheumatology and Immunology, Department of Internal Medicine, Vanderbilt University Medical Center, Tennessee Valley Healthcare System Veterans Affairs Medical Center, Nashville, TN, United States; 4 Division of Rheumatology, UTHealth Houston, Houston, TX, United States

**Keywords:** connective tissue disorder, PDE4B, phosphodiesterase inhibition, progressive fibrosis, systemic sclerosis

## Abstract

Systemic sclerosis (SSc) is a complex autoimmune disease marked by vasculopathy, immune dysregulation, and progressive fibrosis of the skin and internal organs. The high disease burden and impact on quality of life highlight the need for therapies that target underlying pathogenic mechanisms rather than merely treating organ-specific symptoms. Phosphodiesterases, including phosphodiesterase 4B (PDE4B), are key regulators of cellular function and inflammatory responses of several cell types central to SSc pathogenesis. Given PDE4B’s broad effects, targeting it may provide benefits beyond organ-specific manifestations and improve current care. Selective PDE4B inhibition represents a novel therapeutic approach for SSc, with improved subtype selectivity compared with earlier pan-PDE4 inhibitors. Nerandomilast preferentially inhibits PDE4B (ninefold over PDE4D), potentially improving tolerability versus oral pan-PDE4 inhibitors by avoiding adverse events such as gastrointestinal intolerance. Preclinical findings in SSc-relevant mouse and cell models show that PDE4B inhibition modulates inflammatory cytokine secretion, smooth muscle cell tension, endothelial and epithelial barrier integrity, and extracellular matrix synthesis under oxidative stress; this suggests nerandomilast could attenuate immune dysregulation, fibrosis, and potentially impact vasculopathy. Two pivotal Phase III trials evaluated nerandomilast in patients with idiopathic pulmonary fibrosis (FIBRONEER™-IPF; NCT05321069) and progressive pulmonary fibrosis (FIBRONEER™-ILD; NCT05321082), the latter of which included patients with SSc-associated interstitial lung disease (SSc-ILD). The ongoing CONQUEST trial (NCT06195072) is investigating the efficacy and safety of various drugs, including nerandomilast, in patients with early active SSc-ILD. This perspective highlights the rationale for targeting PDE4B in SSc therapy and explores preclinical findings in SSc and clinical findings in SSc-ILD to date.

## 1 Introduction

Systemic sclerosis (SSc) is an autoimmune rheumatic disease characterized by three components: vasculopathy, immune dysregulation, and progressive fibrosis of the skin and several internal organs (1–4). While the specific role of oxidative stress in the pathogenesis of vascular injury remains to be clarified, excess reactive oxidizing species are implicated in the initial or intermediate pathogenesis of SSc, triggering inflammatory cascades and potentially causing endothelial damage (5). Progressive endothelial injury with excessive deposition of collagen in the skin and internal organs can lead to irreversible tissue damage and terminal organ dysfunction (1, 3). Patients with SSc experience a high disease burden, with SSc having the highest morbidity and mortality rates of all the autoimmune rheumatic diseases (3, 4). SSc-associated interstitial lung disease (SSc-ILD) is the current leading cause of SSc-related death and a prevalent complication of SSc, occurring in 30–90% of patients, with 25–30% developing progressive pulmonary fibrosis (PPF) (3, 4, 6). Despite being a major cause of morbidity and mortality in patients with SSc-ILD, the therapies currently approved for SSc-ILD—nintedanib, tocilizumab (US only) and mycophenolate mofetil (Japan only)—do not stop or reverse the fibrotic progression of interstitial lung disease (ILD) once established, representing an important unmet need (1, 7–11).

Current treatment approaches for SSc are largely organ-based, focusing on preserving organ function and managing symptoms rather than addressing the underlying mechanisms driving disease progression (1). For example, recent European Alliance of Associations for Rheumatology recommendations emphasize treating individual SSc-related organ complications rather than targeting the systemic nature of the disease (12). While hematopoietic stem cell transplantation has shown promise in reducing capillary loss and improvement of fibrosis, it should only be considered for carefully selected patients with rapidly progressive SSc and a poor prognosis for survival due to the risk of transplant-related mortality (3, 12, 13). Currently approved pharmaceutical therapies can slow, but do not stop, fibrotic progression and tissue remodeling of the lungs and other organs (1, 12). CD19-targeting chimeric antigen receptor T cells, bispecific T-cell engagers targeting B-cell maturation antigen, CD19, or CD20 might offer great potential as well, but further studies are required to determine the safety and long-term outcomes of these treatments (14–18). Therefore, there remains a significant unmet need for therapies that more broadly prevent, halt, or reverse fibrosis by targeting key underlying pathways (1, 12).

## 2 Phosphodiesterase 4 inhibition and the potential of nerandomilast in SSc

Phosphodiesterases (PDEs) play a critical role in cellular function. Comprising 11 subfamilies (PDE1–PDE11), these enzymes mediate the hydrolysis of cyclic adenosine monophosphate (cAMP) and cyclic guanosine monophosphate, which affects cell proliferation, migration, differentiation, vascular remodeling, secretion of inflammatory cytokines, smooth muscle cell tension, as well as the stability of endothelial and epithelial barriers in lung tissue, all of which are relevant in SSc (19, 20). Among them, PDE4 has been studied most extensively in inflammation. The PDE4 subfamily (PDE4A, PDE4B, PDE4C, and PDE4D) selectively degrades cAMP and plays a vital role in regulating the balance of second messengers in various tissues (21). PDE4 subtypes are widely distributed in the body but have subtype variant-specific tissue distribution (22). PDE4B is the most strongly expressed subtype of PDE4, with highest expression in the lung, spleen, and brain, and is the major subtype of PDE enzyme expressed in immune cells during inflammation (20, 22). Notably, PDE4B is highly expressed in several tissues impacted by SSc, including the lung, immune cells, heart, and skeletal muscle (22), with expression further increased under conditions such as ischemia-reperfusion injury (23).

Nerandomilast is an oral preferential inhibitor of PDE4B with approximately ninefold selectivity for PDE4B versus PDE4D (24). PDE4B mediates the breakdown of cAMP (22, 24), which is a key secondary messenger and a regulator of fibroblast function (**Figure 1A**). As the PDE4B subtype is highly expressed in SSc-relevant tissues (22), it is an attractive target for the treatment of SSc. PDE4B inhibition has the potential to impact the three components of SSc pathogenesis, including targeting inflammation, which in part is vasculopathy-driven, and the early fibrotic stage of SSc (26, 27). Inhibition of PDE4B increases intracellular cAMP, reducing the release of pro-inflammatory mediators and increasing the synthesis of anti-inflammatory cytokines while reducing fibroblast proliferation, myofibroblast differentiation, and extracellular matrix synthesis (22, 24). Increased cAMP also activates exchange proteins, which are directly activated by cAMP 1/2 and protein kinase A (22). Nerandomilast has been shown to induce the dedifferentiation of human lung myofibroblasts (27); however, this has not yet been shown in human dermal fibroblasts. The preferential inhibition of PDE4B by nerandomilast means that effective dosing may be achieved without some of the adverse events (AEs) associated with pan-PDE4 inhibitors (19, 28).

**Figure 1:**
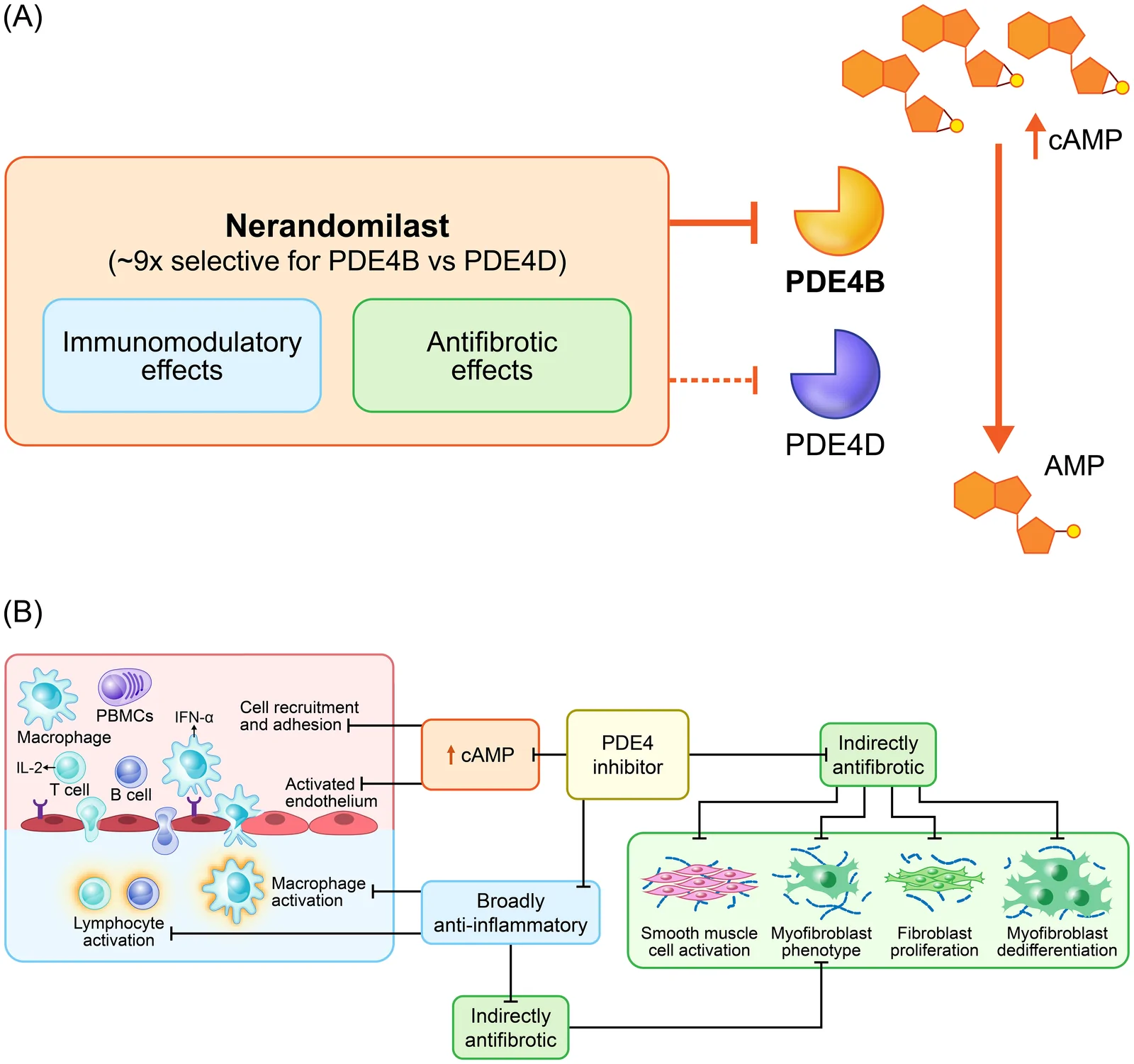
Molecular mechanism of action and pathologic targets of nerandomilast in SSc. **(A)** The PDE4B inhibitor nerandomilast is an oral preferential inhibitor of PDE4B with approximately ninefold selectivity for PDE4B vs PDE4D (24). It promotes the accumulation of intracellular cAMP and has demonstrated both immunomodulatory and antifibrotic effects in preclinical models (25). **(B)** PDE4 inhibition broadly suppresses inflammation by reducing cell recruitment and adhesion to activated endothelium, inhibiting macrophage and lymphocyte activation (20). These anti-inflammatory effects may contribute to downstream antifibrotic effects. Additionally, PDE4 inhibitors may have direct antifibrotic effects, reducing the proliferation of fibroblasts and preventing myofibroblast differentiation (19). AMP, adenosine monophosphate; cAMP, cyclic AMP; IFN-α, type 1 interferon; IL-2, interleukin-2; PBMC, peripheral blood mononuclear cell; PDE, phosphodiesterase; SSc, systemic sclerosis.

## 3 Mechanism of disease and rationale for nerandomilast in SSc

Given that the pathogenesis of SSc is characterized by three components—vasculopathy, immune dysregulation, and fibrosis (**Figure 2**) (1, 29, 30)—broad-acting treatments that interfere with these processes are needed.

**Figure 2:**
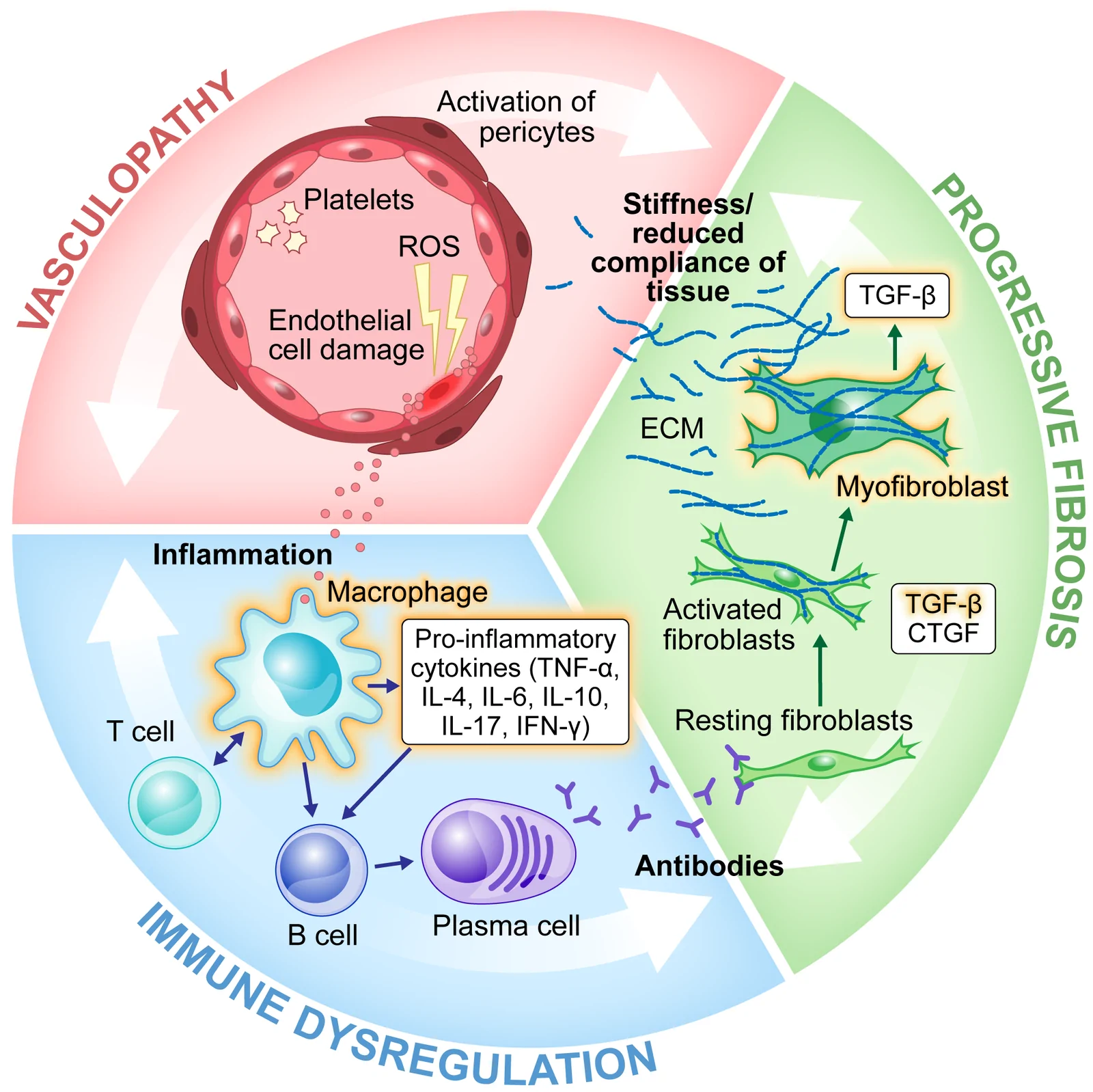
SSc is characterized by a complex interplay between vasculopathy, immune dysregulation, and progressive fibrosis of the skin and several internal organs (34). Injury of the endothelial cells, for example as a result of oxidative stress, leads to local inflammation and activation of immune cells such as T cells, B cells, and macrophages (5, 34). Immune cell activation promotes synthesis of pro-inflammatory cytokines (34). This activates progenitors of ECM-producing myofibroblasts, such as pericytes and resting fibroblasts (2, 34). Differentiation of fibroblasts to myofibroblasts results in tissue remodeling in several internal organs and skin thickening due to excessive collagen deposition (34, 35). In preclinical models, nerandomilast has demonstrated an effect on several of the key pathways implicated in the pathogenesis of SSc (cells/pathways highlighted in yellow). Nerandomilast has immunomodulatory effects on normal human macrophages, inhibiting secretion of key pro-inflammatory cytokines (TNF-α and IL-6) (25). Nerandomilast has also shown antifibrotic effects in SSc patient-derived and healthy dermal fibroblasts stimulated with TGF-β1, a key growth factor in promoting fibroblast-to-myofibroblast differentiation (35). CTGF, connective tissue growth factor; ECM, extracellular matrix; IFN-γ, interferon-γ; IL, interleukin; ROS, reactive oxidizing species; SSc, systemic sclerosis; TGF-β1, transforming growth factor-β1; TNF-α, tumor necrosis factor-α.

Vasculopathy, namely peripheral microvascular abnormalities, is considered the earliest event in SSc pathogenesis (31). Chronic oxidative stress as a result of excess reactive oxidizing species can contribute to endothelial cell damage, leading to recurrent tissue-specific ischemia or reperfusion injury, both of which promote local inflammation and wider immune activation (5). Together, these factors serve as a critical link between autoimmunity and dysregulated fibrosis, establishing a complex interaction and self-perpetuating cycle of inflammation, vascular damage, and progressive fibrosis.

Immune dysregulation in SSc is marked by the involvement of several cytokines that may mediate inflammatory responses in the early stages of SSc, including tumor necrosis factor-α (TNF-α), interleukin (IL)-6, IL-17, IL-4, interferon-γ (IFN-γ), and IL-10 (**Figure 2**) (32, 33, 36). TNF-α may contribute to fibrosis by activating vascular endothelium and promoting fibroblast production of type 1 collagen, thereby driving fibrotic tissue remodeling at least in the more inflammatory stages of SSc (32). PDE4B inhibition reduces the release of pro-inflammatory cytokines, including TNF-α, suggesting potential for mediating any TNF-α-induced immune dysregulation and fibrosis in SSc (19, 22). PDE4B inhibition also reduces the release of the pro-inflammatory cytokine IL-2 and increases the synthesis of anti-inflammatory cytokines by reducing the availability of 5’-AMP as a result of cAMP hydrolysis and increasing cAMP availability (19, 22). The involvement of PDE4 in inflammatory processes and its overexpression in multiple inflammatory diseases, such as psoriasis and atopic dermatitis, have made PDE4 a key pharmacologic target for these diseases (37). PDE4 inhibitors exhibit broad anti-inflammatory activity through suppression of multiple inflammatory cell types, including neutrophils, T cells, and macrophages; this inhibition subsequently reduces cytokine and chemokine release, in addition to antifibrotic activity through suppression of myofibroblast phenotype and fibroblast proliferation (**Figure 1B**) (19).

Uncontrolled production of collagen and extracellular matrix proteins by fibroblasts in the skin, lungs, and other organs results in the accumulation of connective tissue and, therefore, fibrosis (38). Progressive accumulation of connective tissue over extended periods compromises normal tissue architecture, leading to dysfunction and eventual organ failure (38). As such, SSc is characterized by fibrosis of several internal organs (4). Importantly, the fibroblast-to-myofibroblast transition is a key effector step in SSc that could be a therapeutic target (30), and RNA-sequencing evidence suggests that nerandomilast induces myofibroblast dedifferentiation (27).

### 3.1 Clinical experience to date with PDE4 inhibitors

The oral pan-PDE4 inhibitors apremilast and roflumilast, neither of which is approved for the treatment of SSc, have established anti-inflammatory effects, with treatment increasing intracellular cAMP, reducing immune cell activation, and attenuating inflammation in the autoimmune skin condition plaque psoriasis (20, 39–42). In Phase III clinical trials of patients with plaque psoriasis (43, 44) and psoriatic arthritis (45–47), as well as in long-term extension studies (48, 49), oral apremilast demonstrated efficacy and sustained clinical benefit for up to 5 years with a favorable safety profile, improving skin and joint outcomes. Oral roflumilast was also efficacious and safe in treating moderate-to-severe plaque psoriasis over 24 weeks (41, 42). These findings demonstrate the therapeutic relevance of PDE4 inhibition in other immune-mediated diseases. In addition, early clinical trials of PDE4B/D-selective inhibitors such as orismilast have demonstrated anti-inflammatory activity (50, 51) and promising therapeutic benefit for patients with moderate-to-severe plaque psoriasis, with a safety profile consistent with PDE4 inhibition (52), highlighting ongoing developments in this drug class.

The use of oral pan-PDE4 inhibitors has been limited by class-related systemic effects, most notably gastrointestinal AEs such as emesis, believed to result primarily from PDE4D inhibition (53). These AEs frequently lead to treatment discontinuation (19, 22). Gastrointestinal AEs may be particularly problematic in patients with SSc, as gastrointestinal tract involvement is also highly prevalent and contributes substantially to poor quality of life and patient outcomes (54). There are also concerns that apremilast may be associated with increased risk of AEs, including depression (37). Preferential inhibition of the PDE4B subtype may help reduce the risk of such side effects while maintaining beneficial immunomodulatory, antifibrotic, and potential vasoprotective effects.

## 4 Rationale for nerandomilast from preclinical models of SSc

Inhibition of PDE4B by nerandomilast has demonstrated efficacy in preclinical models thought to recapitulate certain features of inflammation, vasculopathy, and fibrosis in SSc (26, 27).

### 4.1 Nerandomilast demonstrates immunomodulatory effects on SSc patient-derived peripheral blood mononuclear cells and normal human macrophages, inhibiting TNF-α and IL-6 secretion

Nerandomilast has demonstrated immunomodulatory effects in several SSc preclinical models when used at clinically relevant concentrations. In cell models of inflammation, treatment with increasing concentrations of nerandomilast (approximately 0.001–20 μM in human whole blood (24) and 0.5 μM in human macrophages (25)) inhibited the release of TNF-α and IL-6, which are key mediators of inflammatory immune dysregulation in the early stages of SSc (24). 0.5 μM nerandomilast in human macrophages also disrupted markers of type 1 IFN signaling (25), a pathway increasingly considered key to SSc pathogenesis. These findings demonstrate that nerandomilast has broad immunomodulatory effects, which could represent a therapeutic advantage compared with therapies targeting individual cytokines. For example, TNF inhibitors such as infliximab and etanercept have not demonstrated consistent clinical benefits in SSc (55–57) and are not recommended for routine use (58), whereas the IL-6 inhibitor tocilizumab has demonstrated clinical effectiveness in slowing decline in lung function (59–61) and may be considered for the treatment of patients with early inflammatory diffuse cutaneous SSc (12, 60). IFN-directed therapies such as anifrolumab may be effective; however, data from Phase III clinical trials are needed to understand their therapeutic potential (62, 63). Other cytokine-directed therapies targeting the IL-2, IL-4, and IL-13 pathways have shown some early efficacy signals in SSc (64, 65), but longer and more comprehensive clinical trials are required to determine clinical relevance.

In normal and SSc patient-derived peripheral blood mononuclear cells exposed to lipopolysaccharide, clinically relevant concentrations of nerandomilast exhibited immunomodulatory effects, inhibiting TNF-α secretion in both normal and SSc patient-derived peripheral blood mononuclear cells compared with the vehicle control (25, 66). This inhibitory effect was maintained in both healthy and SSc patient-derived peripheral blood mononuclear cells when nerandomilast was used in combination with tocilizumab, nintedanib, or mycophenolate mofetil (MMF); however, no effect was seen when tocilizumab, nintedanib, or MMF were used alone (25, 66).

Further evidence for the immunomodulatory effects of nerandomilast was obtained from human macrophages exposed to lipopolysaccharide and prostaglandin E2 (PGE2) in the presence of nerandomilast or MMF (66). PGE2 is a mediator produced during inflammation that enhances IL-6 synthesis, thereby mimicking the inflammatory microenvironment observed in SSc by selectively inducing IL-6 *in vitro* (67, 68). Nerandomilast inhibited TNF-α and IL-6 secretion, but MMF did not significantly affect secretion (66). When macrophages were treated with nerandomilast in combination with MMF, inhibition was similar to treatment with nerandomilast alone (66). Collectively, these results suggest that the immunomodulatory effects of nerandomilast are distinct from those of other treatments and are maintained in combination with standard of care *in vitro* (66).

Nerandomilast may disrupt type 1 IFN signaling, a pathway increasingly recognized as a key driver of SSc pathogenesis (69). Treatment with nerandomilast reduced the expression of the type 1 IFN markers IFN-α-inducible protein 27 and IFN-stimulated gene 15 (66). Additionally, nerandomilast led to a numerical reduction in lipopolysaccharide-induced secretion of the type 2 IFN marker IFN-γ-induced protein 10 (IP-10), also known as CXCL10. In SSc, IP-10 forms part of an IFN-inducible chemokine profile, with proteomic studies demonstrating elevated IP-10 alongside other IFN-γ-inducible proteins. These increases correlate with clinical severity, including skin and lung involvement (70). IFN-γ exposure causes SSc skin fibroblasts to release IP-10, which reinforces inflammation and drives tissue damage (71). These data indicate that IP-10 is a strong marker of disease activity and severity in SSc, with potential to inform clinical management and treatment decisions.

### 4.2 Nerandomilast has antifibrotic effects in fibroblasts and a bleomycin-induced model of fibrosis

Nerandomilast may also impact fibrotic pathways, which are important drivers of organ damage in patients with SSc. Fibroblasts are a heterogeneous population of cells, and their activation to myofibroblasts is a key step in driving the deposition of extracellular matrix, leading to fibrosis (72, 73). Certain fibroblast subpopulations are positively correlated to skin fibrosis, whereas others are linked to a potential antifibrotic role, such as P116+ and CXCL12+ cells (72).

The effects of nerandomilast on the expression of fibrosis markers and extracellular proteins have been assessed in SSc patient-derived dermal fibroblasts stimulated with transforming growth factor-β1 (TGF-β1) and PGE2 to promote fibroblast-to-myofibroblast differentiation (25). Treatment with clinically relevant concentrations of nerandomilast reduced proliferation both alone and in combination with MMF (66). Furthermore, nerandomilast reduced expression of preselected markers associated with fibroblast activation in SSc, such as insulin-like growth factor-binding protein-3, soluble intercellular adhesion molecule-1, and plasminogen activator inhibitor-1 (25).

In the Scar-in-a-Jar assay (74), another model of fibrosis, SSc patient-derived fibroblasts exposed to TGF-β were treated with nerandomilast to assess effects on expression of α-smooth muscle actin (α-SMA) and fibronectin 1 (25, 66). Nerandomilast (0.5 μM) decreased α-SMA levels compared with vehicle control, both in combination with MMF and alone, with similar effects on fibronectin expression (66).

Nerandomilast has also been shown to effectively inhibit skin and lung fibrosis in a bleomycin-induced fibrosis mouse model (26). PDE4B inhibition promoted the accumulation of cAMP in lung tissue, enhancing the phosphorylation of the cAMP response element-binding protein (26). Clinically relevant concentrations of nerandomilast (5 mg/kg twice daily and 12.5 mg/kg twice daily) reduced the expression of profibrotic and pro-inflammatory factors in both lung and skin tissues. Expression of antifibrotic genes (*Acta2*, *Col-1*, *Fn*, and *Ctgf*) was upregulated in the lung tissue of the bleomycin group compared with the control group (26). Nerandomilast dose-dependently reduced the hydroxyproline content and reduced the expression of fibrosis-related markers such as fibronectin, type 1 collagen, and α-SMA in the skin of bleomycin-challenged mice. The nerandomilast-treated mice exhibited alleviated weight loss and reduced mortality rates compared with untreated bleomycin-challenged mice (26). In addition, separate study findings in a bleomycin-induced mouse model show that nerandomilast effectively suppresses the development of fibrosis, with reduced dermal thickening, reduced myofibroblast counts, and reduced hydroxyproline content (data not shown). Although a dose-dependent response was not observed in this study, these results provide additional confidence that the effect of nerandomilast translates well into a mechanistic model of fibrosis *in vivo*.

### 4.3 Nerandomilast induces de-differentiation of myofibroblasts, reducing fibrosis biomarker levels

In human idiopathic pulmonary fibrosis (IPF), lung fibroblasts stimulated with TGF-β1 to induce myofibroblast differentiation, treatment with nerandomilast, or high-concentration PGE2 for 72 hours led to diminished fibroblast contractility as assessed by functional features (27). Additionally, myofibroblast dedifferentiation was observed, as indicated by decreased expression levels of α-SMA, connective tissue growth factor, and plasminogen activator inhibitor-1 (27).

## 5 Nerandomilast clinical data

The Phase III FIBRONEER™-IPF (NCT05321069) and FIBRONEER™-ILD (NCT05321082) trials evaluated oral nerandomilast 18 mg twice daily and 9 mg twice daily in patients with IPF and PPF, respectively. Both trials met their primary endpoint, which was the absolute change from baseline to Week 52 in forced vital capacity (75, 76). In each trial, the curves showing changes in forced vital capacity over time for nerandomilast and placebo separated by Week 2 and continued to diverge up to Week 76 (77, 78), indicating sustained slowing of disease progression with nerandomilast. Nerandomilast demonstrated an acceptable safety and tolerability profile, with diarrhea as the most commonly reported AE, occurring more frequently in patients taking background nintedanib (77, 78). In FIBRONEER™-IPF, the key secondary composite endpoint of time to first acute exacerbation of IPF, hospitalization for respiratory cause, or death over the whole trial was not met with either dose; however, oral nerandomilast 18 mg twice daily was associated with a numerically lower risk of death (77). The FIBRONEER™-ILD trial, which included some patients with SSc as an underlying etiology, showed that nerandomilast reduced the risk of clinically important outcomes, including death, over the whole trial (78). Findings from the subgroup of patients with autoimmune ILD were consistent with the overall trial population in slowing forced vital capacity decline and reducing the risk of death (79). However, this trial did not have SSc-specific endpoints (76). Oral nerandomilast 18 mg twice daily is the recommended dosage that is now approved for the treatment of both IPF and PPF in several countries worldwide (80–85). Patients receiving background antifibrotics (nintedanib and pirfenidone) are also included within the population approved to receive oral nerandomilast 18 mg twice daily; however, in patients who are unable to tolerate 18 mg twice daily, the dosage may be reduced to 9 mg twice daily, except in patients who concomitantly use nerandomilast with pirfenidone (80).

CONQUEST (NCT06195072) is an ongoing Phase IIb platform trial investigating the efficacy, safety, and pharmacodynamics of multiple investigational products, including nerandomilast, for the treatment of early SSc-ILD, and is the first nerandomilast clinical trial with skin and other SSc-relevant endpoints (86). SARD-ILD (NCT06806592) is a Phase IIIb placebo-controlled trial currently in the recruitment phase (87). The objective of this study is to evaluate the efficacy and safety of nerandomilast in improving lung function and fibrotic progression in patients with systemic autoimmune rheumatic disease-associated ILD, including SSc, rheumatoid arthritis, idiopathic inflammatory myopathy, Sjögren’s disease, and mixed connective tissue disease (87).

## 6 Closing opinion

In conclusion, the preferential PDE4B inhibitor nerandomilast demonstrates activity across several key pathogenic pathways in SSc (25–27), providing strong rationale for selectively targeting PDE4B in this disease. Early data suggest that PDE4B inhibition may improve microcirculatory function and mitigate endothelial injury (23), although additional studies are needed to clarify its potential role in SSc-related vasculopathy. Preclinical SSc models further indicate that nerandomilast exerts both immunomodulatory and antifibrotic effects (25, 26), supporting its relevance across the inflammatory and fibrotic dimensions of SSc pathogenesis. A clinical study in early active SSc with ILD (CONQUEST) is currently underway and is expected to yield critical insights into the impact of nerandomilast on clinically meaningful skin-related outcomes. Overall, further investigation into the broader therapeutic effects of nerandomilast across diverse SSc disease manifestations is warranted.

## Data Availability

The raw data supporting the conclusions of this article will be made available by the authors, without undue reservation.
